# Airway segmentation on CT – A systematic review of machine learning tools

**DOI:** 10.1016/j.ejro.2026.100764

**Published:** 2026-05-30

**Authors:** Nada Lsloum, Ahmed Maiter, Turki Alnasser, Ahod Albylwi, Khalid Alghamdi, Michael Sharkey, Alireza Hokmabadi, Mahan Salehi, Krit Dwivedi, Chris Johns, Andrew J. Swift, Samer Alabed

**Affiliations:** a School of Medicine and Population Health, The University of Sheffield, Sheffield, United Kingdom; b Department of Diagnostic Radiology, College of Applied Medical Sciences, Najran University, Najran, Saudi Arabia; c Department of Clinical Radiology, Sheffield Teaching Hospitals, Sheffield, United Kingdom; d Insigneo Institute, Faculty of Engineering, The University of Sheffield, Sheffield, United Kingdom; e National Institute for Health and Care Research, Sheffield Biomedical Research Centre, Sheffield, United Kingdom; f Radiological Sciences Department, College of Applied Medical Sciences, King Saud bin Abdulaziz University for Health Sciences, Riyadh, Saudi Arabia

**Keywords:** Chest CT, Airway, Machine learning, Artificial intelligence, Segmentation, Systematic review

## Abstract

**Background:**

Airway assessment on computed tomography (CT) can yield clinically useful information for diagnosis, treatment planning and monitoring in respiratory diseases. Manual airway segmentation is time-consuming, prone to error and poorly reproducible. This systematic review aimed to appraise machine learning (ML) methods for fully automated airway segmentation in chest CT imaging.

**Methods:**

EMBASE, MEDLINE and CENTRAL were searched on October 28, 2025, for studies which used fully automated ML methods for airway segmentation on CT and reported quantitative performance metrics. The quality of included studies was assessed by the Must AI Criteria-10 (MAIC-10) checklist. PROSPERO [CRD42025635504]

**Results:**

Thirty-two studies (28 used deep learning (DL)) published between 2010 and 2025 were included. Airway segmentation was performed on non-contrast CT scans in most studies. Voxel-wise accuracy metrics were generally high with Dice similarity coefficient (DSC) values ranging between 83% and 96%. Airway-specific topological metrics: branch detection rate (BD) and tree length detection rate (TD) showed broader variability (60–95% and 54–95% respectively), with DL methods consistently outperforming classical ML approaches. Fifteen studies conducted external validation (EXACT’09 test set used in 9/15). MAIC-10 was moderate and ranged from 6 to 8 out of 10, with lowest reporting in safety/privacy (31%), explainability (31%) and transparency (53%).

**Conclusion:**

ML models achieved strong airway segmentation accuracy but showed considerable variation in topological completeness. Standardised evaluation frameworks and the adoption of more diverse datasets are needed to strengthen model generalisability and support translation into clinical practice.

## 1 Introduction

**Fig. 1:**
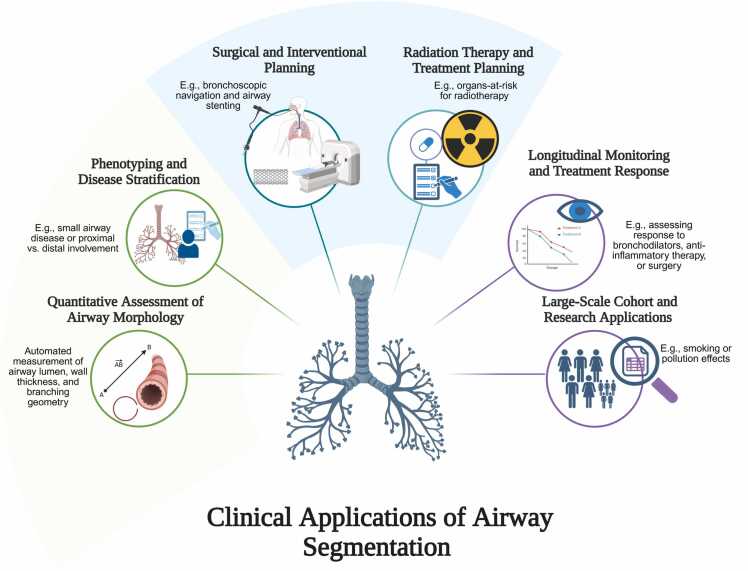
Potential clinical applications of airway segmentation. Created with BioRender.com.

Information about airway structure can support diagnosis, patient risk stratification, surgical planning, and monitoring of severity and treatment response in many respiratory disorders [1], [2], [3], [4], [5] (Fig. 1). Clinically useful metrics - such as wall thickness, luminal diameter and branching geometry - can be derived non-invasively from volumetric computed tomography (CT) following segmentation of the airways [6]. Traditionally, segmentation has been performed manually, a process that is not only time-consuming (taking up to 15 h per scan) [7], but also prone to errors and inter-observer variability [8], [9].

**Fig. 2:**
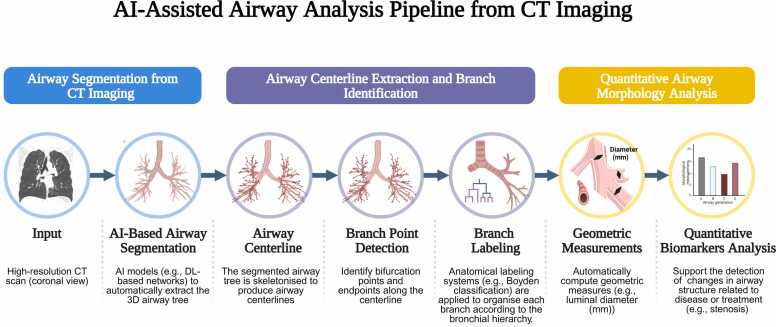
AI-assisted airway analysis pipeline from CT imaging, illustrating the stages of segmentation, centreline extraction, branch identification, and quantitative morphology analysis. The first four conceptual icons are adapted from Tekatli et al. [10] in accordance with the CC-BY licence, while the remaining components were newly designed and created for this review. Created with BioRender.com.

The application of artificial intelligence (AI) for automated airway segmentation on CT has the potential to achieve more accurate, robust, and efficient results [10] (Fig. 2). Prior to the widespread adoption of AI, traditional approaches had been widely employed for airway segmentation [11]. These methods rely on predefined rules and intensity-based operations such as region growing, airway tree reconstruction, and image foresting transform to delineate air-filled structures [12], [13], [14]. While effective for large central airways, these approaches often fail to capture small distal branches due to limited spatial resolution and imaging artifacts [11].

These limitations have motivated the increasing adoption of data-driven machine learning (ML) methods for airway segmentation. Classical ML methods such as k-nearest neighbour (KNN) [15], support vector machines (SVM) [16], and AdaBoost [17] outperformed traditional techniques in accuracy and false-positive reduction. However, these methods typically rely on experts manually designed, hand- crafted image features, such as intensity, texture and shape descriptors, which are time-consuming and often lack robustness in handling complex or variable imaging conditions [18]. More recently, deep learning (DL), a subtype of ML, has become the dominant approach for airway segmentation. By leveraging multiple layers of neural networks, DL can capture more complex features from imaging data, enabling more complete and robust segmentation of the airway tree [18].

This systematic review aims to identify and critically evaluate fully automated ML-based airway segmentation methods in CT imaging to better understand the clinical utility and impact of these tools.

## 2 Materials and methods

This systematic review was conducted and presented in accordance with the Preferred Reporting Items for Systematic Reviews and Meta-Analyses (PRISMA) guidelines [19] (The PRISMA 2020 checklist is provided in Supplementary Table S1). The study protocol was prospectively registered in the International Prospective Register of Systematic Reviews (PROSPERO) under the registration number CRD42025635504.

### 2.1 Search strategy and selection criteria

A comprehensive literature search was performed using the EMBASE and MEDLINE (Ovid) and Cochrane CENTRAL databases on October 28, 2025. The search strategy was designed around three key concepts: artificial intelligence (AI), computed tomography (CT), and the airway tree, including terms related to both normal and diseased airways. The full search strategy is provided in Supplementary Table S2. Studies published online ahead of print at the time of the search were also considered for inclusion.

Studies were deemed eligible for inclusion if they met all the following criteria:

1. Involved majority adult human participants with or without respiratory disease who underwent chest CT with or without intravenous contrast.
2. Used fully automated ML methods for airway segmentation.
3. Reported quantitative metrics of accuracy or other objective measures of AI tool effectiveness.
4. Were peer-reviewed original journal articles published in English.

Studies using traditional non-AI segmentation methods or studies focusing solely on airway classification, detection, or diagnosis without segmenting the airway tree were excluded. Additional exclusions included non-human or non-CT-based studies, manual or semi-automated approaches, non-original publications (including reviews, editorials, conference abstracts, and preprints) and non-English articles. Details of excluded studies and reasons for exclusion are provided in Supplementary Table S3.

### 2.2 Data analysis

**Fig. 3:**
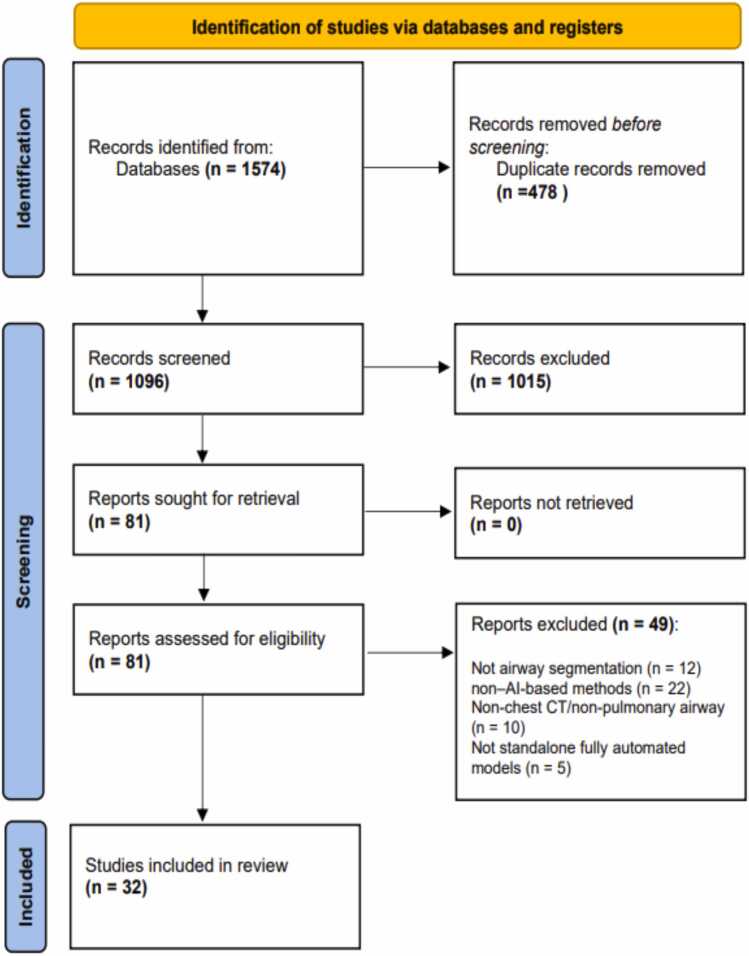
PRISMA 2020 flow diagram illustrating the selection process of studies included in the systematic review [19].

A flow diagram of the study selection process is provided in Fig. 3. A web-based systematic review management tool (Rayyan [20]) was used for deduplication and screening of the records identified from database searching. Screening was carried out in two stages. First, titles and abstracts were assessed to identify potentially eligible studies. Next, the full text articles of the potentially eligible studies were retrieved and assessed against the inclusion criteria. Both stages were conducted independently by two authors (NL and SA) with consensus agreement in cases of uncertainty. The references within eligible studies were manually reviewed to identify any additional relevant articles. Data extraction was performed by one author (NL) using a standardised spreadsheet and was then independently reviewed for accuracy and completeness by two other authors (AA and KA), who cross-checked the extracted data against the full-text articles. Extracted information included study characteristics, patient cohorts (age, sex, and disease or healthy status), CT imaging protocols, AI methodology, and reported performance metrics. The selection of extracted variables was informed by MAIC-10 (Must AI Criteria-10) checklist, which provides a comprehensive guide and standardised criteria for designing and reporting medical imaging AI studies [21].

Six performance metrics were assessed in the included studies: Dice similarity coefficient (DSC), precision, sensitivity, specificity, branch detection rate (BD), and tree length detection rate (TD) (definitions and formulas; Supplementary Table S4). These were grouped into voxel-wise accuracy metrics and topological completeness metrics [11], [22].Voxel-wise accuracy metrics quantify how accurately the segmentation captures airway voxels relative to the reference mask and include DSC (which measures the degree of spatial overlap between predicted and reference airway regions), and sensitivity, specificity and precision (which measure the proportion of predicted airway voxels that are correct). Topological completeness metrics evaluate segmentation of the airway branching structure, independent of voxel-level overlap. They require skeletonizing the airway tree and identifying individual branches and include BD (the percentage of anatomical branches correctly detected relative to the reference tree) and TD (the percentage of the total airway tree length correctly segmented relative to the reference tree).

The quality of included studies was assessed independently by NL and TA using MAIC-10 with consensus opinion used in cases of disagreement (Supplementary Table S5) [21]. MAIC-10 was selected as it is specifically designed for evaluating AI studies in medical imaging, focusing on reporting quality, transparency, and methodological completeness. The included studies primarily represent technical development and retrospective validation of segmentation models rather than diagnostic accuracy studies following a conventional index test–reference standard framework. Therefore, the application of traditional risk-of-bias assessment tools designed for diagnostic accuracy studies is limited in this context. As MAIC-10 does not include a formal risk-of-bias assessment, this was addressed through a narrative discussion of potential sources of bias.

## 3 Results

Thirty-two studies published between 2010 and 2025 were included, including studies available online ahead of print at the time of the search (Fig. 3) [6], [9], [10], [15], [16], [18], [23], [24], [25], [26], [27], [28], [29], [30], [31], [32], [33], [34], [35], [36], [37], [38], [39], [40], [41], [42], [43], [44], [45], [46], [47], [48].

### 3.1 Description of included studies

Across the 32 included studies, the median number of CT scans used for model development was 110, with a range from 40 to 1180 scans. Public datasets were used in 19 studies (59%), private institutional datasets in 9 (28%; 5 single-centre and 4 multi-centre) and both public and private datasets in 4 studies (13%). Most studies (30/32, 94%) used retrospectively collected datasets for training and evaluation of their proposed models, while 6% (2/32) employed prospectively collected data. DL was the predominant AI approach, applied in 87% (28/32) of studies, while the remaining 13% (4/32) used ML methods (Table 1). Detailed characteristics of included studies and AI models’ architectures are provided in Supplementary Table S6.

**Table 1:** Characteristics of the Included Studies (n = 32).

**Deep Learning–based Studies (n = 28)**							
**Author, Year**	**Cohort**	**CT scans**	**Age (mean ± SD year)**	**Sex (Female%)**	**Framework**	**Datasets Used (number of CTs)**	
						**Training and Internal Validation**	**External Testing**
Wang 2026 [23]	Mixed (healthy, fibrosis, and COVID-19)	512	NR	NR	U-Net	ATM’22: 200	AIIB23: 312
Pang 2025 [24]	NR	90	NR	NR	U-Net	BAS: 90	NA
Zhao 2025 [25]	Mixed (healthy and COPD)	388	NR	NR	U-Net	Private: 25 EXACT’09: 20 BAS: 90 Private: 243	ISICDM2021: 10
Liu et 2025 [26]	Mixed (healthy, non-COPD and COPD)	593	Healthy: 51 ± 8 Mild COPD: 73 ± 11 Severe COPD**:**71 ± 9	Healthy: 30 (41%) Mild COPD: 45 (28%) Severe COPD:50 (28%)	U-Net	Private: 413	Private: 180
Zhou 2025 [27]	NR	70	NR	NR	U-Net	ATM’22: 70	NA
Wang 2025 [28]	NR	418	NR	NR	U-Net	ATM’22: 298 AIIB23: 120	NA
Zhang and Gu 2024 [29]	NR	110	NR	NR	U-Net	BAS: 90	EXACT’09: 20
Støverud 2024 [30]	Mixed (healthy and mixed pulmonary disease)	327	AeroPath: 69 ± 8*	AeroPath: 9 (33%)	U-Net	ATM’22: 300	AeroPath: 27
Zhu 2024 [31]	NR	90	NR	NR	CNN	LIDC: 70 EXACT’09: 20	NA
Zhang 2024 [18]	NR	300	NR	NR	U-Net	ATM’22; 300	NA
Tekatli 2024 [10]	SABR patients	59	Internal: 66 ± 12* External:68 ± 12 *	Internal: 8/26 (31%) External: 18/33 (55%)	CNN	Private: 26	Private: 33
Nan 2024 [32]	Mixed (healthy and diseased: lung cancer, COVID-19, and pulmonary fibrosis)	140	NR	NR	CNN	BAS: 90	Private: 50
Yuan 2024 [9]	NR	67	NR	NR	CNN	ISICDM 2020: 67	NA
Wu 2023 [33]	COPD and COVID 19	1180	Private 2: 64 ± 12	Private 2: 219 (52%)	U-Net	EXACT’09: 20 BAS: 70 ATM’22: 349 Private1: 23 Private 2: 420	EXACT’09: 20 ISICDM2021: 12 BAS: 20 ATM’22: 150 RICORD: 96
Zhang 2023 [34]	Mixed (healthy and mixed pulmonary disease)	90	NR	NR	U-Net	LIDC-IDRI: 70 EXACT’09: 20	NA
Dudurych 2023 [35]	Mixed (healthy and intermediate lung nodules)	400	60 ± 9	70/ 168 (42%)	U-Net	ImaLife: 400	NA
Khanna 2023 [36]	NR	40	NR	NR	U-Net	VESSEL12: 20 EXACT’09: 20	NA
Ke 2023 [37]	NR	60	NR	NR	U-Net	BAS: 60	NA
Weikert 2022 [38]	Mixed (healthy and COPD)	575	68 ± 10	202 (35%)	U-Net	Private: 575	NA
Guo 2022 [39]	NR	40	NR	NR	CNN	Private: 20 EXACT’09: 20	NA
Zheng 2021 [40]	NR	110	NR	NR	U-Net	BAS: 90	EXACT’09: 20
Garcia-Uceda 2021 [41]	Mixed (healthy and severe pulmonary disease)	76	CF-CT: 12 ± 3* DLCST:60 ± 5*	CF-CT: 10 (42%)	U-Net	CF-CT: 24 DLCST: 32	EXACT’09: 20
Qin 2021 [42]	NR	110	NR	NR	CNN	LIDC-IDRI: 70 EXACT’09: 20	EXACT’09:20
Cheng 2021 [43]	Mixed pulmonary diseases	120	52 ± 17*	NR	CNN	Private: 100	EXACT'09: 20
Zhou 2021 [6]	Mixed (healthy and diseased)	250	NR	NR	CNN	Private: 250	NA
Nadeem 2021 [44]	Mixed (healthy and COPD)	172	Data_stand:_ 63 ± 10 Data_repro:_ 30 ± 11 Data_low:_ 35 ± 12	Data_stand:_ 60 (50%) Data_repro:_ 10 (50%) Data_low:_ 5 (42%)	U-Net	SPIROMICS, Data_stand,_: 120 Private, Data_repro_: 40 Private, Data_low_: 12	NA
Selvan 2020 [45]	NR	132	NR	NR	Graph-based DL (MFN + GNN)	DLCST: 132	NA
Yun 2019 [46]	Mixed (healthy and COPD)	97	NR	NR	CNN	KOLD: 77	EXACT’09: 20

**Machine Learning–based Studies (n = 4)**							
Lee 2019 [47]	COPD	85	NR	NR	SVM	KOLD: 65	EXACT’09: 20
Bian 2018 [48]	COPD	100	NR	NR	RFC	COPDGene: 100	NA
Meng 2017 [16]	NR	50	NR	NR	SVM	Private: 50	NA
Lo 2010 [15]	Lung cancer and emphysema	270	60 ± 5*	NR	KNN	DLCST: 250	EXACT’09: 20

**Table note.** Studies are listed by publication date (2026 → 2010). * Indicates age values were reported as median (range) or range only and were converted to mean ± SD for consistency. CT scans refer to the total number of scans used in each study, including training, validation, and external testing datasets. Detailed characteristics of included studies and public datasets are provided in Supplementary Tables S6 and S7.

**Abbreviations:** NR, not reported; NA, not applicable; CT, computed tomography; SD, standard deviation; COVID-19, coronavirus disease 2019; COPD, chronic obstructive pulmonary disease; SABR, stereotactic ablative radiotherapy; U-Net, encoder–decoder convolutional neural network for biomedical image segmentation; CNN, convolutional neural network; DL, deep learning; MFN, mean-field network; GNN, graph neural network; SVM, support vector machine; RFC, random forest classifier; KNN, k-nearest neighbours; BAS, Binary Airway Segmentation dataset; EXACT’09, Extraction of Airways from CT Challenge 2009; ATM’22, Airway Tree Modelling Challenge 2022; AIIB23, Airway-Informed Imaging Biomarker 2023; ISICDM, International Symposium on Image Computing and Digital Medicine; RICORD, RSNA International COVID-19 Open Radiology Database; AeroPath, Pathologically Challenging Airway Dataset; LIDC-IDRI, Lung Image Database Consortium and Image Database Resource Initiative; ImaLife, Imaging in Lifelines; KOLD, Korean Obstructive Lung Disease Cohort; SPIROMICS, SubPopulations and InteRmediate Outcome Measures In COPD Study; COPDGene, Genetic Epidemiology of Chronic Obstructive Pulmonary Disease study; VESSEL12, VESsel SEgmentation in the Lung (2012 Challenge); DLCST, Danish Lung Cancer Screening Trial; CF-CT, cystic fibrosis CT; Datastand, standard-dose SPIROMICS total lung capacity chest CT dataset; Datarepro, reproducibility dataset with repeat total lung capacity chest CT scans; Datalow, low-dose total lung capacity chest CT dataset.

For validation, 17 studies (53%) used internal validation only, while 15 (47%) employed both internal and external validation. Internal validation involved held-out subsets or cross-validation within the training data, while external validation used completely independent datasets. Most studies (9/15, 60%) used the EXACT’09 (Extraction of Airways from CT Challenge 2009) public dataset for external testing [11]. None of the airway segmentation methods identified are used in routine clinical practice or have regulatory approval (Table 1; Supplementary Table S6**)**.

21 of 32 studies (66%) reported the type of CT acquisition used. Among these, (19/21) utilised non-contrast CT (NCCT) scans, which included various types such as low-dose CT, high resolution CT (HRCT), and standard-dose volumetric chest CT. The remaining two studies reported the use of contrast-enhanced CT (CECT) imaging. Importantly, both explicitly used arterial CT angiography (CTA) rather than routine CECT. Støverud et al. [30] used CTA images in their testing dataset (AeroPath), while Yuan et al. [9] included a mixed dataset comprising 43 CTA and 24 NCCT scans used for training, validation and testing. Neither study provided explicit details on contrast dose or injection timing.

12 of 32 studies (38%) reported detailed CT acquisition parameters. Among these, the majority (6/12, 50%) used Siemens SOMATOM scanners. Reported tube voltages ranged from 100 to 140 kVp, while tube currents varied widely between 20 and 200 mAs, reflecting both low and standard-dose protocols. Slice thickness also varied from 0.4 to 2.0 mm, with most scans reconstructed at 0.5–1.25 mm.

Patient characteristics were infrequently reported (10/32 studies, 31%) with a pooled mean age 63 ± 3 years ranging approximately 18–88 years; one study incorporated a small subset (n = 24/76, 31%) of paediatric cystic fibrosis dataset (6–17 years) [41]. Where reported, females accounted for overall 40% (726/1814) of the study population. Ethnicity was not reported in any study. Cohort descriptions were reported in 18/32 studies (56%), most included mixed healthy and diseased populations, while chronic obstructive pulmonary disease (COPD)/emphysema was the most frequently represented pathology (10/18, 56%) (Table 1). However, information on disease diagnosis and severity was poorly reported (9/32 studies, 28%).

Airway segmentation was the primary objective in most studies (24/32, 75%), while others incorporated it either alongside additional segmentation tasks (3/32, 9%) [31], [34], [42], or as part of multi-organ or downstream analytical pipelines (5/32, 16%) [10], [24], [26], [35], [38].

The reference standard annotations were clearly defined in most studies (30 of 32 studies, 94%) and were generated through manual or semi-automatic annotations by radiologists using contouring tools such as ITK-SNAP, Mimics, or in-house editors. Several (15 of 30) studies used multi-expert annotation workflows, including consensus review, multi-round adjudication, or validation by senior thoracic radiologists. Some studies relied on radiologist annotations provided in public datasets. No study reported inter- or intra-observer variability for the reference standard segmentation.

### 3.2 Performance evaluation of airway segmentation methods

**Fig. 4:**
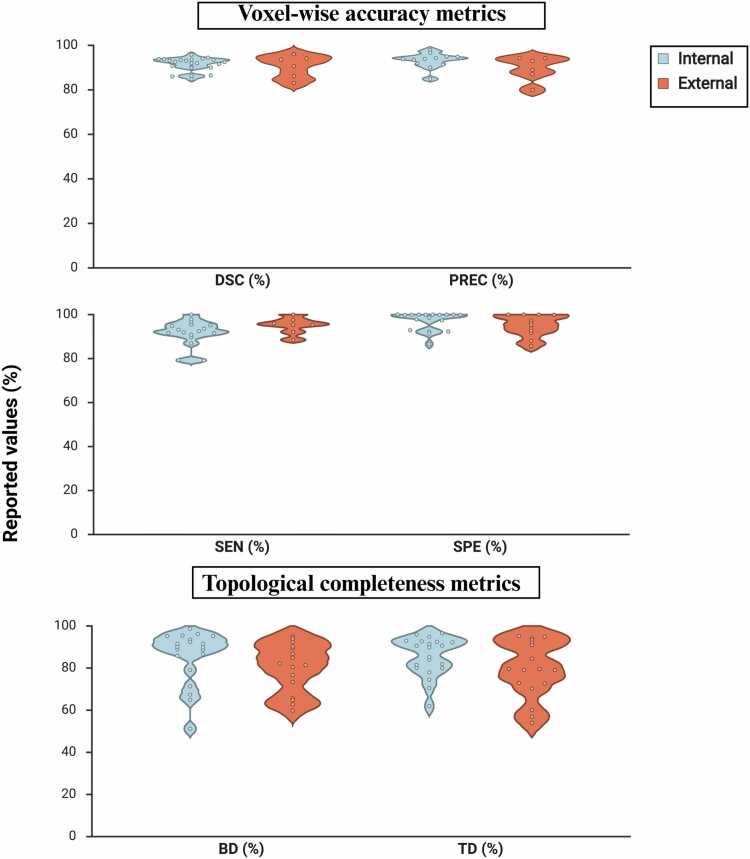
Distribution of airway segmentation performance metrics across the included studies. Voxel-wise accuracy metrics: Dice Similarity Coefficient (DSC), Precision (PREC), Sensitivity (SEN), and Specificity (SPE) and topological completeness metrics: Branch Detection (BD) and Tree Length Detection (TD). All metrics are reported as percentages. For each metric, results from internal validation and external testing are shown side by side. Violin plots illustrate the distribution of reported study-level values, with dots represent individual study results. Created with BioRender.com.

Given the heterogeneity across studies in cohorts, reference standards and validation strategies, formal quantitative pooling or meta-analysis was not appropriate. Thus, performance metrics are summarised using minimum–maximum ranges. The values of the six-performance metrics are presented in Table 2 and Fig. 4.

**Table 2:** Quantitative Metrics of Airway Segmentation Performance from Included Studies (%).

		**Voxel wise accuracy metrics**				**Topological metrics**	
**Author, Year**	**Internal/ External validation (dataset used or model tested)**	**DSC%**	**PREC%**	**SEN%**	**SPE%**	**BD%**	**TD%**
Wang 2026 [23]	Internal	93.8	-	91.6	100	93.5	89.8
	External	93.6	-	88.5	100	62.8	72.9
Pang 2025 [24]	Internal	92.0	-	-	-	85.9	90.6
Zhao 2025 [25]	Internal (BAS)	-	84.9	-	-	95.2	94.9
	External	-	-	96.0	-	94.9	95.2
Zhou 2025 [27]	Internal	93.8	97.9	-	-	92.5	95.9
Wang 2025 [28]	Internal (ATM’22)	93.1	-	93.1^†^	99.9	71.3	80.1
	Internal (AIIB23)	90.8	-	89.7^†^	99.9	51.2	62.0
Zhang and Gu 2024 [29]	Internal	-	-	-	100	91.5	96.5
	External	-	-	-	93.7	82.1	79.6
Støverud 2024 [30]	Internal	89.9	93.9	100.0	86.6	64.9	79.9
	External	83.1	80.0	100.0	88.0	89.4	94.1
Zhu 2024 [31]	Internal	-	90.0	-	-	91.5	93.0
Zhang 2024 [18]	Internal	92.6	93.5	91.9	99.9	-	-
Nan 2024 [32]	Internal	93.3*	91.9	94.8^‡^	-	89.0	92.7
	External (25/50 COVID-19)	96.0*	94.3	97.6^‡^	-	90.2	93.3
	External (25/50 Fibrosis)	90.6*	89.0	92.1^‡^	-	73.4	79.0
Yuan 2024 [9]	Internal	93.1	93.9	92.5	-	67.4	78.0
Wu 2023 [33]	External (ISICDM 2021)	86.1	-	95.4	100	94.0	92.1
	External (EXACT’09)	-	-	-	91.7	81.4	79.6
	External (BAS)	-	87.0	-	-	92.4	94.9
	External (ATM’22)	94.1	93.0	95.3	100	86.7	91.0
Zhang 2023 [34]	Internal	93.6	-	91.6	-	95.5	84.0
Dudurych 2023 [35]	Internal	92.0	-	-	92.9	-	-
Khanna 2023 [36]	Internal **(**VESSEL12 & EXACT’09)	94.1	-	95.5	-	-	-
Ke 2023 [37]	Internal	94.5	94.3	95.2	-	90.1	91.4
Weikert 2022 [38]	Internal	86.0	-	86.9	-	-	-
Guo 2022 [39]	Internal **(**Private)	93.5	96.7	90.8	100	-	-
	Internal (EXACT’09)	95.8	95.0	96.6	99.9	-	-
Zheng 2021 [40]	Internal	-	91.4	-	-	88.7	92.5
	External	-	94.2	-	-	80.5	79.0
Garcia-Uceda 2021 [41]	Internal	91.6	-	-	-	-	81.5
	External	-	-	-	97.3	-	70.3
Qin 2021 [42]	Internal	92.5	-	93.6	100	96.2	90.7
	External	-	-	-	96.3	76.7	72.7
Cheng 2021 [43]	Internal	90.3	-	-	98.6	86.6	-
	External	-	-	-	85.7	84.9	84.5
Zhou 2021 [6]	Internal	86.2	94.9	79.3	100	-	-
Nadeem 2021 [44]	Internal (Standard dose)	-	-	-	-	95.2	-
	Internal (Low dose)	-	-	-	-	98.7	-
Selvan 2020 [45]	Internal (MFN)^§^	86.5	-	-	91.4	-	74.5
	Internal (GNN)^§^	84.8	-	-	92.2	-	81.9
Yun 2019 [46]	Internal	90.0	-	-	92.3	89.8	92.2
	External	-	-	-	95.4	65.7	60.1
Lee 2019 [47]	Internal	-	-	-	97.8	-	70.5
	External	-	-	-	92.7	64.9	56.9
Bian 2018 [48]	Internal	-	-	-	97.4	**-**	**-**
Meng 2017 [16]	Internal	-	-	79.1	99.9	79.1	85.2
Lo 2010 [15]	Internal	-	-	97.8		-	-
	External	-		-	99.9	59.8	54.0

**Table note.** Performance metrics are reported separately for internal or external validation, as specified in each study. All values are reported as percentages (%) and rounded to one decimal place. “–” indicates not reported. Specificity values were derived from the false positive rate for consistency across studies.

* Intersection over Union converted to Dice using the standard formula: *Dice* = (2 × *IoU*) / (1 + *IoU*).

† Sensitivity derived from false negative rate using the formula: *Sensitivity* (%) = (1 −*FNR*) × 100.

‡ Sensitivity derived from airway missing ratio using the formula: *Sensitivity* (%) = (1 −*AMR*) × 100

§ Two graph-based models (a mean-field network and a graph neural network) were evaluated and statistically compared using internal eight-fold cross-validation.

**Abbreviations:** DSC, Dice similarity coefficient; PREC, precision; SEN, sensitivity; SPE, specificity; BD, branch detection; TD, tree detection; BAS, Binary Airway Segmentation dataset; ATM’22, Airway Tree Modeling Challenge 2022; AIIB23, Airway-Informed Imaging Biomarker 2023; COVID-19, coronavirus disease 2019; ISICDM, International Symposium on Image Computing and Digital Medicine; EXACT’09, Extraction of Airways from CT Challenge 2009; VESSEL12, VESsel SEgmentation in the Lung (2012 Challenge); MFN, mean-field network; GNN, graph neural network.

Voxel-wise segmentation performance was generally high for both internal validation and external testing. Across studies, internal validation showed DSC values of 86–96%, sensitivity of 79–100%, specificity of 87–100%, and precision of 85–98%, while topological metrics were more variable (BD: 51–99%; TD: 62–97%). External testing demonstrated similarly strong voxel-wise performance (DSC: 83–96%; sensitivity: 89–100%; specificity: 86–100%; precision: 80–94%), with wider variability in BD (60–95%) and TD (54–95%). The nine studies using the EXACT’09 dataset for external testing were fairly compared in Table 3.

**Table 3:** Comparative Performance of AI Models Evaluated on the EXACT’09 External Test Set (n = 9 Studies).

**Author, Year**	**SPE %**	**BD%**	**TD%**
Zhang and Gu 2024 [29]	93.7	82.1	79.6
Wu 2023 [33]	91.7	81.4	79.6
Zheng 2021 [40]	94.2	80.5	79.0
Garcia-Uceda 2021 [41]	97.3	-	70.3
Qin 2021 [42]	96.3	76.7	72.7
Cheng 2021 [43]	85.7	**84.9**	**84.5**
Yun 2019 [46]	95.4	65.7	60.1
Lee 2019 [47]	92.7	64.9	56.9
Lo et 2010 [15]	**99.9**	59.8	54.0

**Table note.** Comparative performance of AI-based airway segmentation models externally evaluated on the EXACT’09 challenge test set (20 CT scans). Performance metrics include specificity, branch detection rate, and tree-length detection rate. “–” indicates not reported. The highest value for each performance metric is highlighted in bold.

**Abbreviations:** SPE, specificity; BD, branch detection rate; TD, tree-length detection rate.

Two studies used clinically relevant outcomes as performance metrics. Tekatli et al. [10] demonstrated earlier and improved airway-stenosis detection compared with visual inspection (58% vs. 31%), and Liu et al. [26] assessed COPD diagnosis performance (AUC 0.96; sensitivity 97%; specificity 92%). Wu et al. [33] further applied their airway segmentation model to clinical COPD and COVID-19 datasets using reference-independent airway biomarkers (e.g., branch count, tree length, airway volume). These measures showed significant reductions with increasing COPD severity and in COVID-19.

### 3.3 Quality of reporting

**Fig. 5:**
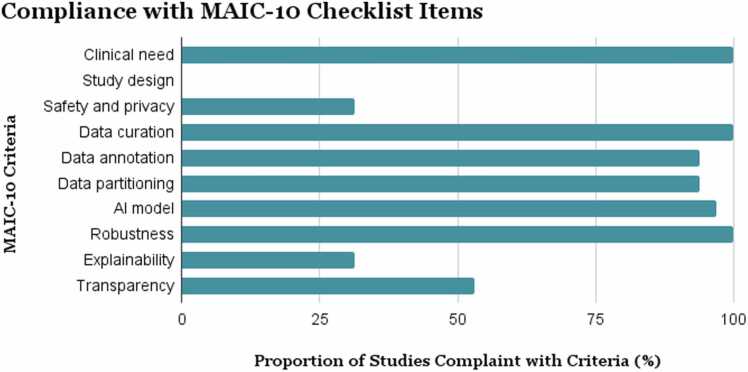
Summary of adherence to the MAIC-10 criteria across the included studies.

Compliance with MAIC-10 is summarised in Fig. 5. Detailed descriptions and study-level adherence to MAIC-10 criteria are provided in the Supplementary Material (Supplementary Table S5).To maintain consistency and ensure fair cross-study evaluation, the high BD and TD values from Bian et al. [48] were not included in the quantitative comparison in Table 2, as they were computed against incomplete reference annotations that missed distal airways.

## 4 Discussion

This systematic review presents a comprehensive overview of 32 studies employing fully automated ML-based methods for airway segmentation on CT imaging. The results demonstrate clear trends in study design, methodological approaches, performance metrics and quality of reporting.

Mirroring the wider field of AI for medical imaging, most studies used DL techniques with convolutional neural network (CNN) architectures. Such architectures, including variations of U-Net, are particularly effective in medical image segmentation tasks due to their ability to capture pertinent image features from data in an end-to-end optimised framework, making them adaptable to varying anatomical structures and imaging modalities beyond the airway tree [49], [50]. Our study supports a shift toward end-to-end CNN architectures for medical imaging segmentation [18].

NCCT was the commonest type of CT acquisition for airway segmentation. NCCT is widely performed for lung and airway imaging and can incorporate high-resolution and low-dose techniques [51], [52]. The anatomical structure of the airway lumen is clearly characterised on NCCT images due to the inherent difference in attenuation between air within the lumen and the surrounding airway walls. Only two studies used contrast-enhanced CT; the impact of intravenous contrast on model performance remains unclear, but no clear performance advantage was observed [30]. Intravenous contrast is typically administered to improve soft tissue differentiation, and it is unknown whether this could improve segmentation performance in the context of airway pathology, for example [53], [54], [55].

In addition to the general accuracy performance metrics, reviewing the topological metrics of BD and TD provided deeper insight into the anatomical completeness of the segmented airway trees. According to the ATM’22 (Airway Tree Modelling Challenge 2022), these metrics are crucial in identifying models that perform well beyond voxel-wise overlap, capturing more important clinical details [22]. Despite generally high segmentation accuracy scores, there was considerable variability in topological metrics, suggesting that many models struggle with segmenting peripheral airway branches. This was particularly noted among the classical ML-based models, such as the KNN classifier and the SVM approach used by [15] and [47], which reported the lowest TD and BD values (Table 2, Table 3). Benchmark results from EXACT’09 and ATM’22 show that DL models demonstrated superior performance in airway topological continuity compared with classical ML methods, achieving approximately 90% in BD and TD scores, whereas no classical method in the EXACT’09 benchmark exceeded 74% total tree length [22]. This is likely due to the reliance of classical ML methods on handcrafted features and predefined rules, which are insufficient to capture the complex geometry and high variability of peripheral airway structures. In contrast, DL models can learn hierarchical spatial representations directly from imaging data, enabling improved detection of distal airway branches. This result underscores the limitations of classical ML approaches in capturing the peripheral airway as well as further emphasises the suitability of advanced DL methods in airway segmentation [11], [22]. In this context, human-in-the-loop approaches that iteratively retrain models on corrected failed or suboptimal segmentations may offer a practical strategy for improving performance, particularly for peripheral airway branches [56].

The review also highlights inconsistencies in evaluation metrics and reference standard annotation strategies adopted across studies. As presented in Table 2, some studies focus on general performance metrics [6], [18], [39], while ignoring airway-specific topological metrics: BD and TD despite their importance in this context. Importantly, the clinical implications of airway segmentation models, particularly in settings like bronchoscopy planning, post-treatment airway assessment, or quantifying airway disease burden, depends not just on general overlap metrics like DSC, but on the accurate identification of peripheral airway branches. Higher BD reflects improved detection of distal airways, which is especially relevant in diseases such as COPD, where small airway involvement is prominent. Similarly, TD reflects the completeness of airway tree reconstruction, which is critical for navigation and procedural planning. Under-segmentation of these branches may lead to incomplete visualisation of diseased segments, potentially impacting clinical decision-making or treatment planning.

In terms of reference standard quality, the omission of inter- and intra-observer variability reporting across all studies limits assessment of annotation reliability and uncertainty. In addition, reference standard completeness varied across studies, particularly for peripheral airways which might misrepresent true model performance (e.g., Bian et al. [48]).

A key determinant of clinical applicability for AI-based airway segmentation models is their ability to generalise across different patient populations, imaging conditions, and healthcare settings [57]. Several studies demonstrated substantial performance degradation when models were evaluated on more clinically diverse datasets [23], [28], [41], [42], [46], [47] (Fig. 4). For example, models proposed by Wang et al. [23] Zhang and Gu [29] and Yun et al. [46] reported reduced performance, especially in topological metrics, when tested on external cohorts with severe lung disease compared to their performance on internal or cleaner datasets. Such decreases in performance can also reflect model overfitting to specific datasets, underscoring the importance of external validation and the use of diverse, multi-institutional data to improve generalisability.

A further important observation emerged from the EXACT’09 comparison (Table 3), where the methods proposed by Zhang and Gu [29], Wu et al. [33], and Cheng et al. [43] achieved among the highest topological completeness scores while recording relatively lower specificity. This pattern suggests that some approaches may sacrifice voxel-wise accuracy in order to capture a greater number of airway branches. A similar trade-off between topological completeness and correctness was observed in the ATM’22 challenge, where several models demonstrated comparable behaviour. Consequently, the ATM’22 challenge emphasised the need for careful handling of correctness while maintaining high topological completeness [22]. Reduced specificity on EXACT’09 may also be partly attributable to incomplete distal airway annotations in the reference standard, resulting in some true predictions being incorrectly labelled as false positives.

Public datasets (Supplementary Table S7) were used in different stages of model development and evaluation by most included studies. Particularly, 22 studies used public datasets for training and internal validation, while 12 studies exclusively relied on them for external validation. Despite their importance in airway segmentation research including facilitating reproducibility, transparency, and comparability across studies, as well as access to large and well-annotated data, commonly used public benchmark datasets also present important limitations. For example, EXACT’09 is constrained by challenge-oriented, task-specific reference standards that do not fully capture fine-grained airway anatomy, while ATM’22 is limited to screening and relatively homogeneous populations, restricting its representation of complex clinical pathologies [11], [22]. Additionally, the repeated use of the same benchmark datasets across the development and evaluation of multiple AI models increases the risk of overfitting and benchmarking saturation, whereby models demonstrate strong performance on shared test sets but fail to generalise to real-world clinical scenarios [58]. Public datasets may also carry institutional or regional biases that limit their global applicability and clinical transferability [59], [60]. To mitigate these issues, future research should prioritise the development and use of more diverse, multi-centre, and pathology-rich datasets that better reflect clinical heterogeneity and enable more robust model validation [61].

As noted elsewhere in the field of AI for medical imaging, we found variable quality of reporting [62], [63], [64], [65]. Notably, details about patient cohort characteristics - such as age, sex, pathologies and disease severity - were infrequently reported. This considerably limits the interpretation of model performance, generalisability and clinical utility. Furthermore, none of the included studies provided a rationale or calculation of their chosen sample sizes despite considerable variation (ranging from 40 to 1180 CT scans). Even when constrained using public datasets with predefined sample sizes, these omissions raise concerns regarding the reliability and generalisability of the proposed AI models [21], [66], [67]. Improved adherence to reporting standards such as the Checklist for Artificial Intelligence in Medical Imaging (CLAIM) and MAIC-10 would enhance the consistency, reproducibility, and comparability of airway segmentation research.

### 4.1 Translation into clinical practice

Although none of the reviewed airway segmentation models are currently implemented in routine clinical practice or have obtained regulatory approval, several demonstrated clear clinical potential. For example, quantitative airway morphology analysis including measurements of airway lumen and wall parameters to support diagnosis, phenotyping, severity staging/classification, and monitoring of airway disease such as COPD [25], [26], [35], [38]. In addition, enhanced detection of peripheral bronchi and synthetic CECT generation from NCCT were leveraged for 3D bronchoscopic navigation and treatment planning [34], [40]. ML-based airway segmentation has also shown superior sensitivity compared with expert visual assessment, detecting post-stereotactic ablative radiotherapy (SABR) airway stenosis nearly twice as frequently at earlier time points [10]. These capabilities further support early detection and longitudinal monitoring of radiation-induced airway toxicity following SABR, facilitating evaluation of treatment-related airway injury and potential refinement of radiation dose constraints [10]. Fig. 1 summarises potential clinical applications of airway segmentation.

Several of the ML-based airway segmentation models are publicly available, presenting an important step toward reproducibility, benchmarking, and future clinical translation [18], [24], [33], [40], [41], [45]. However, despite the overall promising advances in segmentation accuracy and topological completeness, all the reviewed ML models were evaluated retrospectively, indicating that they represent technical validation rather than prospective clinical or diagnostic accuracy studies, further limiting the applicability of conventional risk-of-bias tools. Prospective evaluation under real-world conditions is required before they can be translated to routine clinical practice.

### 4.2 Limitations of this systematic review

Despite its comprehensive scope, this systematic review has several limitations. First, it focused exclusively on fully automated ML-based airway segmentation tools using CT imaging, and the inclusion criteria were restricted to peer-reviewed articles. This may have introduced publication bias by excluding unpublished studies, such as conference abstracts and preprints. Second, substantial heterogeneity in performance metrics, reference standard annotations, and inconsistent reporting of cohort characteristics limited the ability to perform a formal meta-analysis and prevented direct statistical comparisons between studies. Instead, ranges of performance metrics were presented to descriptively characterise variability across studies without implying direct comparability or statistical pooling. Another limitation is that most models were developed using NCCT and public datasets, while clinical workflows frequently involve CECT and diverse pathologies, highlighting a potential domain gap between research datasets and clinical practice. This discrepancy may limit the generalisability of current models. Finally, although we applied a structured quality assessment tool, the evaluation process involved a degree of subjectivity, particularly in interpreting reporting quality and methodological transparency.

## 5 Conclusion

This systematic review included 32 studies on fully automated ML-based airway segmentation in CT imaging. Trends in technical approaches, performance metrics, dataset usage and reporting practices were assessed. The systematic review highlights the prevalence of DL methods, the added value of topological metrics in testing performance especially in more distal airways, and the popularity of publicly available challenge datasets in model training and testing. Considerable heterogeneity was encountered amongst the studies, as well as important shortfalls in quality of reporting, including inadequate description of patient characteristics.

Future work should focus on standardising performance metrics, using more diverse and clinically representative datasets, and enabling prospective evaluation in real-world clinical settings to support clinical translation. In particular, external validation across multi-institutional cohorts, including CECT acquisitions, is required to address domain shift and improve generalisability. Adoption of benchmarking frameworks such as EXACT’09 and ATM’22 would ensure consistent metric definitions and fair comparison across studies. Future studies should also improve reporting of key dataset and methodological details, including patient demographics, disease distribution, reference standard annotation and preprocessing pipelines. Closer adherence to reporting guidelines such as CLAIM and MAIC-10 is essential to enhance transparency, comparability, and reproducibility of AI research in airway segmentation.

## CRediT authorship contribution statement

**Nada Lsloum:** Writing – review & editing, Writing – original draft, Conceptualization. **Ahmed Maiter:** Writing – review & editing. **Turki Alnasser:** Writing – review & editing, Validation. **Ahod Albylwi:** Writing – review & editing, Data curation. **Khalid Alghamdi:** Writing – review & editing, Data curation. **Michael Sharkey:** Writing – review & editing. **Alireza Hokmabadi:** Writing – review & editing. **Mahan Salehi:** Writing – review & editing. **Krit Dwivedi:** Writing – review & editing. **Chris Johns:** Writing – review & editing. **Andrew J. Swift:** Writing – review & editing, Supervision. **Samer Alabed:** Writing – review & editing, Supervision.

## Ethics statement

This systematic review did not involve human participants or animals. All data were obtained from previously published studies; therefore, ethical approval and informed consent were not required.

## Declaration of Competing Interest

The authors declare that they have no known competing financial interests or personal relationships that could have appeared to influence the work reported in this paper.
