# Supplementary material for: Airway segmentation on CT – A systematic review of machine learning tools
Source: Eur J Radiol Open. 2026 May 30;16:100764. doi: 10.1016/j.ejro.2026.100764 (PMC13240772; doi:10.1016/j.ejro.2026.100764)
Supplement: Supplementary file 1 — Supplementary material [file mmc1.pdf]

Supplementary materials

Supplementary Table S1. PRISMA 2020 checklist

Section and Topic	Item #	Checklist item	Location where item is reported
**TITLE**			
Title	1	Identify the report as a systematic review.	Title page
**ABSTRACT**			
Abstract	2	See the PRISMA 2020 for Abstracts checklist.	Title page
**INTRODUCTION**			
Rationale	3	Describe the rationale for the review in the context of existing knowledge.	Introduction
Objectives	4	Provide an explicit statement of the objective(s) or question(s) the review addresses.	Introduction (Last sentence)
**METHODS**			
Eligibility criteria	5	Specify the inclusion and exclusion criteria for the review and how studies were grouped for the syntheses.	Materials and methods – section 2.1
Information sources	6	Specify all databases, registers, websites, organisations, reference lists and other sources searched or consulted to identify studies. Specify the date when each source was last searched or consulted.	Materials and methods – section 2.1&Fig 3
Search strategy	7	Present the full search strategies for all databases, registers and websites, including any filters and limits used.	Fig 3& Supplementary Table S2
Selection process	8	Specify the methods used to decide whether a study met the inclusion criteria of the review, including how many reviewers screened each record and each report retrieved, whether they worked independently, and if applicable, details of automation tools used in the process.	Materials and methods -section 2.2
Data collection process	9	Specify the methods used to collect data from reports, including how many reviewers collected data from each report, whether they worked independently, any processes for obtaining or confirming data from study investigators, and if applicable, details of automation tools used in the process.	Materials and methods -section 2.2
Data items	10a	List and define all outcomes for which data were sought. Specify whether all results that were compatible with each outcome domain in each study were sought (e.g. for all measures, time points, analyses), and if not, the methods used to decide which results to collect.	Materials and methods -section 2.2, Table 1& Table 2
	10b	List and define all other variables for which data were sought (e.g. participant and intervention characteristics, funding sources). Describe any assumptions made about any missing or unclear information.	Materials and methods -section 2.2, Table 1, Table 2, Supplementary Table S4 &S6
Study risk of bias assessment	11	Specify the methods used to assess risk of bias in the included studies, including details of the tool(s) used, how many reviewers assessed each study and whether they worked independently, and if applicable, details of automation tools used in the process.	Materials and methods -section 2.2
Effect measures	12	Specify for each outcome the effect measure(s) (e.g. risk ratio, mean difference) used in the synthesis or presentation of results.	Materials and methods -section 2.2 & Supplementary Table S4

Section and Topic	Item #	Checklist item	Location where item is reported
Synthesis methods	13a	Describe the processes used to decide which studies were eligible for each synthesis (e.g. tabulating the study intervention characteristics and comparing against the planned groups for each synthesis (item #5)).	Materials and methods -section 2.2
	13b	Describe any methods required to prepare the data for presentation or synthesis, such as handling of missing summary statistics, or data conversions.	NA (No meta-analysis)
	13c	Describe any methods used to tabulate or visually display results of individual studies and syntheses.	NA
	13d	Describe any methods used to synthesize results and provide a rationale for the choice(s). If meta-analysis was performed, describe the model(s), method(s) to identify the presence and extent of statistical heterogeneity, and software package(s) used.	NA
	13e	Describe any methods used to explore possible causes of heterogeneity among study results (e.g. subgroup analysis, meta-regression).	NA
	13f	Describe any sensitivity analyses conducted to assess robustness of the synthesized results.	NA
Reporting bias assessment	14	Describe any methods used to assess risk of bias due to missing results in a synthesis (arising from reporting biases).	NA
Certainty assessment	15	Describe any methods used to assess certainty (or confidence) in the body of evidence for an outcome.	NA
**RESULTS**			
Study selection	16a	Describe the results of the search and selection process, from the number of records identified in the search to the number of studies included in the review, ideally using a flow diagram.	Fig.3
	16b	Cite studies that might appear to meet the inclusion criteria, but which were excluded, and explain why they were excluded.	Supplementary Table S3
Study characteristics	17	Cite each included study and present its characteristics.	Table1& Supplementary Table S6
Risk of bias in studies	18	Present assessments of risk of bias for each included study.	Result – section 3.3 & Fig.5 & Supplementary Table S5
Results of individual studies	19	For all outcomes, present, for each study: (a) summary statistics for each group (where appropriate) and (b) an effect estimates and its precision (e.g. confidence/credible interval), ideally using structured tables or plots.	Table 2, Table 3 &Fig.4
Results of syntheses	20a	For each synthesis, briefly summarise the characteristics and risk of bias among contributing studies.	NA (No meta-analysis)
	20b	Present results of all statistical syntheses conducted. If meta-analysis was done, present for each the summary estimate and its precision (e.g. confidence/credible interval) and measures of statistical heterogeneity. If comparing groups, describe the direction of the effect.	NA
	20c	Present results of all investigations of possible causes of heterogeneity among study results.	NA
	20d	Present results of all sensitivity analyses conducted to assess the robustness of the synthesized results.	NA
Reporting biases	21	Present assessments of risk of bias due to missing results (arising from reporting biases) for each synthesis assessed.	NA
Certainty of evidence	22	Present assessments of certainty (or confidence) in the body of evidence for each outcome assessed.	NA
**DISCUSSION**			
Discussion	23a	Provide a general interpretation of the results in the context of other evidence.	Discussion
	23b	Discuss any limitations of the evidence included in the review.	Discussion
	23c	Discuss any limitations of the review processes used.	Limitations of this systematic

Section and Topic	Item #	Checklist item	Location where item is reported
			review
	23d	Discuss implications of the results for practice, policy, and future research.	Translation into clinical practice
**OTHER INFORMATION**			
Registration and protocol	24a	Provide registration information for the review, including register name and registration number, or state that the review was not registered.	Materials and methods
	24b	Indicate where the review protocol can be accessed, or state that a protocol was not prepared.	Materials and methods
	24c	Describe and explain any amendments to information provided at registration or in the protocol.	NA
Support	25	Describe sources of financial or non-financial support for the review, and the role of the funders or sponsors in the review.	Funding statement
Competing interests	26	Declare any competing interests of review authors.	Declaration of competing interest
Availability of data, code and other materials	27	Report which of the following are publicly available and where they can be found: template data collection forms; data extracted from included studies; data used for all analyses; analytic code; any other materials used in the review.	MAIC-10 checklist / Supplementary materials

From: Page MJ, McKenzie JE, Bossuyt PM, Boutron I, Hoffmann TC, Mulrow CD, et al. The PRISMA 2020 statement: an updated guideline for reporting systematic reviews. BMJ 2021;372:n71. doi: 10.1136/bmj.n71. This work is licensed under CC BY 4.0. To view a copy of this license, visit https://creativecommons.org/licenses/by/4.0/

Supplementary Table S2. Full search strategy

#	**Searches**
1	("Artificial Intelligence" or "Neural Networks" or ((deep or machine or supervised or unsupervised) adj1 learning) or Segmentation
2	*artificial intelligence/
3	*machine learning/
4	*deep learning/
5	*convolutional neural network/
6	2 or 3 or 4 or 5
7	1 or 6
8	*computer assisted tomography/
9	compute* tomograph*
10	8 or 9
11	(airway or respirator* tract or trache* or bronch*)
12	(asthma or COPD or cystic fibrosis or air trapping)
13	*respiratory tract disease/
14	11 or 12 or 13
15	7 and 10 and 14
16	remove duplicates from 15

Supplementary Table S3. Excluded studies and reasons for exclusion

Excluded study (Author, year)	Reason for exclusion
Dole 2025 [1]	Focused on airway obstruction classification rather than airway segmentation.
Mirus 2025 [2]	Focused on lung segmentation and clinical outcome prediction rather than airway segmentation.
Rabby 2025 [3]	Focused on COPD classification using CT imaging rather than airway segmentation
Ma 2025 [4]	Employed semi-automated airway segmentation for radiomic analysis but did not propose or evaluate an airway segmentation method.
Hu 2024 [5]	Relied on classical algorithmic segmentation methods instead of machine learning or deep learning–based airway segmentation
Wang 2024 [6]	Employed a semi-automated deep learning approach involving human-in-the-loop active learning.
Ebrahimian 2023 [7]	Used commercial software for tracheal segmentation rather than an AI-trained segmentation model.
Yu 2023 [8]	Focused on airway anatomical labelling rather than airway segmentation.
Ratwani 2023 [9]	Used manual, operator-dependent segmentation using volume rendering techniques rather than AI-based airway segmentation.
Nadeem 2022 [10]	Focused on airway detection, not full airway segmentation.
Cho 2022 [11]	Used cone-beam CT (CBCT) imaging of the head and focused on pharyngeal airway segmentation rather than thoracic airway segmentation from chest CT.
Boubnovski 2022 [12]	Focused on pulmonary lobar segmentation, not airway segmentation.
Orhan 2022 [13]	Used CBCT scans and focused on craniomaxillofacial anatomy and pharyngeal airway evaluation rather than pulmonary airway segmentation from CT.
Dudurych 2021 [14]	Dataset preparation / training refinement. Primarily focused on dataset creation and manual correction for training purposes, rather than proposing or validating a standalone airway segmentation model.
Zhang 2021 [15]	Focused on segmentation of tracheobronchial diverticula, not general airway segmentation.
Shujaat 2021 [16]	Used CBCT imaging and focused on pharyngeal airway segmentation rather than pulmonary airway segmentation from chest CT.
Ram 2021 [17]	Focused on AI-based detection of air trapping rather than airway segmentation.
Leonardi 2021 [18]	Used CBCT imaging and focused on segmentation of the sinonasal cavity and pharyngeal airway rather than pulmonary airway segmentation from chest CT.

Duan 2020 [19]	Employed traditional image processing techniques (region growing, morphology, and skeleton analysis) rather than AI-based segmentation methods.
Fischer 2020 [20]	Focused on AI-based lung lobe segmentation and emphysema quantification rather than airway segmentation.
Park 2020 [21]	Focused on automated lung lobe segmentation rather than airway segmentation.
Khanna 2020 [22]	Used a rule-based, non–AI approach for airway segmentation, including region growing, morphological reconstruction, and heuristic seed point detection
Sousa 2019 [23]	Used classical image processing techniques based on the Image Foresting Transform (IFT) rather than AI-based airway segmentation.
Gil 2019 [24]	Used traditional structural analysis and adaptive thresholding for airway segmentation.
Selvan 2019 [25]	Employed a multiple hypothesis tracking (MHT) and template-matching approach based on statistical ranking, rather than a deep learning or AI-based segmentation model.
Price 2019 [26]	Focused on geometric validation using paediatric airway phantoms and optical coherence tomography (aOCT), without the use of AI-based airway segmentation.
Charbonnier 2017 [27]	Proposed a post-processing method to refine already segmented airways by detecting leaks; did not perform full airway segmentation.
Neelapu 2017 [28]	Focused on pharyngeal and sinonasal airway segmentation using CBCT, not pulmonary airway segmentation.
Lim 2016 [29]	Focused on lung lobe segmentation and emphysema quantification, not airway segmentation.
Bernardeschi 2016 [30]	Used rule-based and classical image-processing techniques rather than AI.
Xu 2015 [31]	Employed a hybrid image-processing framework for airway wall measurement rather than an AI-based segmentation model.
Pu 2015 [32]	Used geometric shape descriptors and rule-based methods rather than AI or deep learning.
Nardelli 2015 [33]	Used semi-automatic segmentation with classical image processing and manual tuning; no AI-based model employed.
Fabijanska 2015 [34]	Focused on pulmonary vascular tree segmentation, not airway segmentation.
Fetita 2014 [35]	Used deformable active surface models (classical computer vision), not AI.
Heydarian 2014 [36]	Applied morphological image processing for airway analysis without AI.
Petersen 2014 [37]	Used advanced image-processing and surface modelling techniques rather than AI.
de Water 2014 [38]	Used Dolphin 3D software on CBCT scans in paediatric subjects; not AI-based.
Mesanovic 2013 [39]	Used classical image processing techniques for airway segmentation without AI or deep learning.
Liu 2013 [40]	Used graph-search–based segmentation and phantom validation, not AI.

Lassen 2013 [41]	Focused on pulmonary lobe segmentation using classical methods, not airway segmentation.
Chiang 2012 [42]	Used CBCT and commercial software for upper airway analysis, not AI.
Pu 2012 [43]	Used geometric airway analysis without AI or learning-based segmentation.
Cheung 2012 [44]	CBCT-based airway evaluation in paediatric subjects; not AI-based.
Castillo 2012 [45]	Multi-modality study (CT + SPECT) using semi-automated segmentation, not AI.
Barbosa 2011 [46]	Used handcrafted feature analysis for air trapping and emphysema, not AI.
Petersen 2011 [47]	Used graph-based segmentation techniques rather than AI.
Pu 2011 [48]	Used differential geometric modelling, not machine learning or deep learning.
Graham 2010 [49]	Relied on graph-based segmentation for bronchoscopy guidance, not AI.

Supplementary Table S4. Definitions and formulas of voxel-wise and topological performance metrics used for airway segmentation evaluation.

Metric name	Definition	Formula
**Voxel-wise Accuracy Metrics**		
**Dice Similarity Coefficient (DSC)**	Measures spatial overlap between the predicted and reference airway regions.	$DSC = \frac{2TP}{2TP + FP + FN} \times 100\%$
**Precision**	Proportion of predicted airway voxels that are correctly labelled.	$Precision = \frac{TP}{TP + FP} \times 100\%$
**Sensitivity / True Positive Rate (TPR)**	Proportion of true airway voxels that are correctly segmented.	$Sensitivity = \frac{TP}{TP + FN} \times 100\%$
**Specificity**	Proportion of background voxels correctly identified as non-airway; complementary to False Positive Rate (FPR)	$Specificity = 100 - FPR$
**False Positive Rate (FPR)**	Proportion of background voxels incorrectly labelled as airway.	$FPR = \frac{FP}{FP + TN} \times 100\%$
**Topological Completeness Metrics**		
**Branch Detection Rate (BD)**	Percentage of anatomical airway branches correctly identified relative to the reference tree.	$BD = \frac{Nseg}{Nref} \times 100\%$
**Tree Length Detection (TD)**	Percentage of the total reference airway tree length correctly segmented.	$TD = \frac{Lseg}{Lref} \times 100\%$

Abbreviations: TP, true positive; FP, false positive; FN, false negative; TN, true negative; Nseg, number of correctly detected branches; Nref, total number of reference branches; Lseg, length of correctly segmented airway branches; Lref, total reference airway tree length.

Supplementary Table S5. A detailed compliance of the included studies with the MAIC-10 criteria

The included studies demonstrated moderate adherence to reporting standards, with total scores ranging from 6/10 to 8/10. All studies (100.0%) clearly defined a clinical need, described their data curation processes, and addressed robustness, including explicit discussions on the generalisability of the AI models in real-world conditions even when it is limited. The majority of studies also reported on data annotation (93.8%), data partitioning (93.8%), and AI model building methodology (96.9%). However, several key areas were consistently underreported. Only 31.3% of studies addressed safety and privacy considerations, while explainability and transparency were fulfilled by just 31.3% and 53.1% of studies, respectively. Above all, none of the studies fully met the study design criterion, with sample size estimation being the most omitted component.

Study	Clinical Need	Study Design	Safety & Privacy	Data Curation	Data Annotation	Data Partitioning	AI Model	Robustness	Explainability	Transparency	MAIC-QS (/10)
Wang 2026	1	0	0	1	1	1	1	1	0	1	7
Pang 2025	1	0	0	1	0	0	1	1	1	1	6
Zhao 2025	1	0	1	1	1	1	1	1	0	1	8
Liu et 2025	1	0	1	1	1	1	1	1	0	1	8
Zhou 2025	1	0	0	1	1	1	1	1	1	1	8
Wang 2025	1	0	0	1	1	1	1	1	1	1	8
Zhang and Gu 2024	1	0	0	1	1	1	1	1	0	1	7
Støverud 2024	1	0	1	1	1	1	0	1	1	1	8
Zhu 2024	1	0	0	1	1	1	1	1	1	0	7
Zhang 2024	1	0	0	1	1	1	1	1	0	1	7
Tekatli 2024	1	0	1	1	1	0	1	1	0	0	6
Nan 2024	1	0	0	1	1	1	1	1	1	0	7
Yuan 2024	1	0	0	1	0	1	1	1	0	1	6
Wu 2023	1	0	1	1	1	1	1	1	0	1	8
Zhang 2023	1	0	1	1	1	1	1	1	0	0	7
Dudurych 2023	1	0	0	1	1	1	1	1	0	1	7
Khanna 2023	1	0	0	1	1	1	1	1	0	0	6
Ke 2023	1	0	0	1	1	1	1	1	1	1	8
Weikert 2022	1	0	1	1	1	1	1	1	1	0	8
Guo 2022	1	0	0	1	1	1	1	1	0	0	6
Zheng 2021	1	0	0	1	1	1	1	1	0	1	7
Garcia-Uceda 2021	1	0	0	1	1	1	1	1	0	1	7
Qin 2021	1	0	0	1	1	1	1	1	1	1	8
Cheng 2021	1	0	0	1	1	1	1	1	0	0	6
Zhou 2021	1	0	1	1	1	1	1	1	0	0	7
Nadeem 2021	1	0	0	1	1	1	1	1	0	0	6
Selvan 2020	1	0	0	1	1	1	1	1	0	1	7
Yun 2019	1	0	1	1	1	1	1	1	0	0	7

Lee 2019	1	0	0	1	1	1	1	1	0	0	6
Bian 2018	1	0	0	1	1	1	1	1	1	0	7
Meng 2017	1	0	1	1	1	1	1	1	0	0	7
Lo 2010	1	0	0	1	1	1	1	1	0	0	6

Supplementary Table S6. Detailed characteristics of included studies and AI models' architectures

Study Characteristics			Datasets and validation		AI models architectures		
**Author, Year**	**Country**	**Study Design**	**Dataset**	**Validation**	**Architecture**	**Architecture - Details**	**Network Dimension**
Wang 2026	China	Retrospective	Public	Internal + external validation	Multi-task DL framework	U-Net–based CNN (multi-task, dual-branch) framework that jointly predicts airway mask (reconstruction) and airway centerline, with a two-branch shared backbone based on over-complete representation and LISB for fine detail.	3D
Pang 2025	China	Retrospective	Public	Internal validation	SegTom	An end-to-end 3D volumetric medical image **Seg**mentation framework on thoracoabdominal multi-organ ana**tom**ical structure	3D
Zhao 2025	China	Retrospective	Multiple public	Internal + external validation	DCT-UNet	Dilated Contextual -UNet	3D
Liu et 2025	China	Retrospective	Private, single centre	Internal + external validation	Multi-stage DL pipeline	nnU-Net for airway segmentation → centerline & arc-length → C- (elliptical fitting of inner/outer walls) → quantitative metrics → SVM for COPD classification; Random Forest for exacerbation-related factor importance	3D
Zhou 2025	China	Retrospective	Public	Internal validation	TASTE:3D-UNet–based multi-stage	TASTE — Triple-Attention with Weighted Skeletonised Tversky Loss (WSTL)	3D
Wang 2025	China	Retrospective	Multiple public	Internal validation	UDADS-Net	UDADS-Net — Uncertainty-based Double Attention Detail-Supplement Network targeting distal bronchi. 3D U-Net with attention enhancements	3D
Zhang and Gu 2024	China	Retrospective	Public	Internal + external validation	Connectivity-aware CNN with topology-sensitive loss functions	Modified 3D U-Net with two auxiliary supervision modules: Connectivity-Aware Surrogate loss (CAS) and Local-Sensitive Distance loss (LSD); end-to-end training.	3D

Støverud 2024	Norway	Retrospective	Public	Internal + external validation	AGU-Net	Attention-Gated U-Net with skip-connection attention gates to enhance distal airway segmentation and preserve airway topology	3D
Zhu 2024	Japan	Retrospective	Public	Internal validation	SG-CNN	Skeleton-Guided 3D Convolutional Neural Network	3D
Zhang 2024	China	Retrospective	Public	Internal validation	3D-UNet	A detail-sensitive 3D-UNet (DS-3D-UNet)	3D
Tekatli 2024	Netherlands	Retrospective	Private, single centre	Internal + external validation	MEDPSeg	A Public end-to-end DL model trained on a large, heterogeneous CT dataset for pulmonary structures, including airways	3D
Nan 2024	UK	Retrospective	Public	Internal + external validation	FANN	Fuzzy Attention Neural Network. The model integrates fuzzy logic with attention mechanisms within a CNN framework to improve feature representation and enhance airway continuity in segmentation tasks.	3D
Yuan 2024	China	Retrospective	Public	Internal validation	2D FA-SegNet + 3D ARNet	2D full airway SegNet +3D airway RefineNet	2D+3D
Wu 2023	China	Retrospective	Multiple public + Private, single centre	Internal + external validation	Two-stage 3D segmentation pipeline with Contextual Transformer (CoT) + deep supervision;	Modified 3D U-Net (encoder–decoder) with 3D CoT blocks inserted in encoder & decoder; two-stage training (Stage 1 overall airway; Stage 2 intrapulmonary/segmental–subsegmental); deep supervision heads.	3D
Zhang 2023	China	Retrospective	Multiple public	Internal validation	3D DDU-Net	Distributed dense U-Net/ Amulti-task framework. The model combines distributed dense connections in a multi-task collaborative learning setup for airway and artery-vein segmentation and CECT synthesis	3D
Dudurych 2023	Netherlands	Retrospective	Private, multi-centre	Internal validation	3D U-Net	Bronchinet and Opfront methods (3D-Unet and 3D optimal-surface graph-cut methods)	3D
Khanna 2023	India	Retrospective	Multiple public	Internal validation	U-Net	A convolutional neural network based on deep U-Net	2D
Ke 2023	China	Retrospective	Public	Internal validation	Modified UNet++	UNet++ backbone + Attentional Context Supervision (ACS) + Adaptive Tversky Loss (ATL); 3D convolutions; attention modules integrated	3D

Weikert 2022	Switzerland	Retrospective	Private, single centre	Internal validation	Retina U-Nets	3D U-Net + 2D U-Net pipeline: 3D U-Net (centerline detection) + 2D U-Net (wall segmentation)	3D+ 2D
Guo 2022	China	Retrospective	Public + Private, single centre	Internal validation	Mif-CNN	Multi-information fusion CNN integrates atrous spatial pyramid pooling and fused boundary/location context, and a CNN-based region-growing voxel classification network refines small branches.	3D
Zheng 2021	China	Retrospective	Public	Internal + external validation	WingsNet	UNet with Group Supervision. WingsNet integrates group supervision into an encoder-decoder (U-Net-like) network to provide complementary gradient flows and enhance learning of small airway branches under severe class imbalance	3D
Garcia-Uceda 2021	Netherlands	Retrospective	Public + Private, single centre	Internal + external validation	3D U-Net	A simple and low-memory 3D U-Net as backbone	3D
Qin 2021	China	Retrospective	Multiple Public	Internal + external validation	CNN	A tubule-sensitive CNNs-based method for pulmonary airway and artery-vein segmentation.	3D
Cheng 2021	China	Retrospective	Private, multi-centre	Internal + external validation	TACNet	An end-to-end Tiny Atrous Convolutional Network (TACNet) based on 3D convolution neural network	3D
Zhou 2021	China	Retrospective	Private, single centre	Internal validation	MFA-Net	Multi-scale feature aggregation network	3D
Nadeem 2021	United States	Retrospective	Public + Private, multi-centre	Internal validation	DL-FG	DL-based freeze-and-grow. A 3D encoder–decoder CNN with skip connections is used to predict airway likelihoods and guide a freeze-and-grow propagation algorithm for robust airway tree segmentation.	3D
Selvan 2020	Denmark	Retrospective	Public	Internal validation	MFN and GNN	A graph-based DL framework that refines an initial 3D airway graph using Mean-Field Networks and Graph Neural Networks, learning to correct false positives and recover missing airway branches.	3D
Yun 2019	South Korea	Prospective	Private, multi-centre	Internal + external validation	2.5D CNN	A 2.5D CNN that processes orthogonal or neighbouring CT slices to incorporate limited 3D contextual information while maintaining 2D convolutional efficiency for fully automated airway segmentation.	2.5

Lee 2019	South Korea	Prospective	Private, multi-centre	Internal + external validation	SVM	Hybrid approach (multi-scale filtering + Support Vector Machine (SVM))	3D
Bian 2018	Netherlands	Retrospective	Public	Internal validation	RFC	Random forest classifier. A ML–based approach that extracts hand-crafted features from 3D CT scans and applies a random forest classifier for voxel-level segmentation of small airways.	3D
Meng 2017	Japan	Retrospective	Private, single centre	Internal validation	SVM	Support Vector Machine for false positive removal. A hybrid approach combining local intensity filtering to detect candidate airway voxels, followed by a 3D feature-based SVM for false positive removal and accurate airway segmentation.	3D
Lo 2010	Netherlands	Retrospective	Public	Internal + external validation	KNN	A voxel-wise k-Nearest Neighbours classifier guided by vessel proximity to segment the airway tree from 3D CT volumes.	3D

Abbreviations: AI, artificial intelligence; DL, deep learning; ML, machine learning; CNN, convolutional neural network; U-Net, encoder–decoder convolutional neural network; nnU-Net, no-new-Net U-Net framework; DCT-UNet, dilated contextual U-Net; TASTE, triple-attention with weighted skeletonised Tversky loss; WSTL, weighted skeletonised Tversky loss; UDADS-Net, uncertainty-based double attention detail-supplement network; AGU-Net, attention-gated U-Net; SG-CNN, skeleton-guided convolutional neural network; FANN, fuzzy attention neural network; FA-SegNet, full airway SegNet; ARNet, airway RefineNet; CoT, contextual transformer; DDU-Net, distributed dense U-Net; MEDPSeg, medical pulmonary structure segmentation network; Mif-CNN, multi-information fusion convolutional neural network; MFA-Net, multi-scale feature aggregation network; DL-FG, deep learning freeze-and-grow; MFN, mean-field network; GNN, graph neural network; SVM, support vector machine; RFC, random forest classifier; KNN, k-nearest neighbours; CAS, connectivity-aware surrogate loss; LSD, local-sensitive distance loss; ATL, adaptive Tversky loss; ACS, attentional context supervision; COPD, chronic obstructive pulmonary disease; CT, computed tomography; 2D, two-dimensional; 2.5D, two-and-a-half-dimensional; 3D, three-dimensional

Supplementary Table S7. Overview of public datasets used across the included studies

Dataset	Year	No. of CT scans	Main pathology / population	Centre type	contributing centres	Access (open/ controlled)
**EXACT'09** [50]	2009	40	Healthy volunteers and patients with severe lung disease.	Multi-centre	International challenge dataset	Controlled access (registration/approval required)
**ATM'22** [51]	2022	500	Healthy subjects and COVID-19 CTs with ground-glass opacity and consolidation	Multi-centre (international)	Airway Tree Modelling Challenge contributors	Open
**BAS** [52]	2020	90	Mixed pulmonary cohort; airway-focused benchmark (healthy subjects and patients with pulmonary nodules; diagnoses not explicitly stratified)	Multi-centre	Derived from LIDC-IDRI (7 U.S. academic centres) and EXACT'09 challenge dataset	Open
**AIIB23** [53]	2023	312	Fibrotic lung disease (n = 262) and COVID-19 pneumonia (n = 50)	Multi-centre (international)	Contributed by multiple international institutions as part of the AIIB23 challenge; individual centres anonymised	Controlled access via challenge registration
**ISICDM** [54]	2020–2021	12	Mixed pulmonary cohort (airway-focused benchmark; diagnoses not explicitly specified)	Multi-centre	Contributed by participating institutions of the International Symposium on Image Computing and Digital Medicine (centres not individually disclosed)	Controlled access via challenge registration
**AeroPath** [55]	2024	27	Patients undergoing diagnostic evaluation for lung cancer, with heterogeneous pulmonary pathologies including	Single centre	St. Olavs Hospital, Trondheim, Norway	Open

			malignant tumours, sarcoidosis, and emphysema			
**RICORD** [56]	2020	240	COVID-19	Multi-centre (international)	Four contributing institutions across different countries (centres anonymised by RSNA)	Open
**LIDC-IDRI** [57]	2011	1,018	Thoracic CT scans with annotated lung nodules with mixed pulmonary findings	Multi-centre (USA)	Seven U.S. academic medical centres and eight medical imaging companies	Open
**DLCST** [58]	2009	2,052 baseline CT scans (subset often used in imaging studies)	Current and former heavy smokers (≥20 pack-years) undergoing lung cancer screening; lung nodules and lung cancer cases	Single centre (screening trial)	Gentofte University Hospital, Copenhagen, Denmark	Controlled access (request/ approval required)
**COPDGene** [59]	2010	10,000 participants (example studies often use subsets, e.g., 100 CT scans)	Current and former smokers with and without COPD across GOLD stages; CT phenotypes include emphysema, gas trapping, and airway wall thickening	Multi-centre (USA)	Multiple U.S. academic medical centres participating in the COPDGene consortium; imaging core analysis by Thirona (Netherlands)	Controlled access (request/ approval required)
**VESSEL12** [60]	2012	20	Thoracic CT scans with mixed lung diseases, designed for vessel segmentation; airway masks derived by expert guidance	Multi-centre (challenge dataset)	Contributed by multiple institutions participating in the ISBI 2012 VESSEL12 Challenge (centres not individually disclosed)	Open
**SPIROMICS** [61]	2013	~ 3000	COPD, smokers and non-smokers (with and without COPD) to understand COPD subtypes, mechanisms, and clinically meaningful biomarkers.	Multi-centre	Multiple U.S. clinical centres.	Controlled access (request/ approval required)

Abbreviations: EXACT'09, Extraction of Airways from CT Challenge 2009; ATM'22, Airway Tree Modelling Challenge 2022; CT, computed tomography; COVID-19, coronavirus disease 2019; BAS, binary airway segmentation dataset; LIDC-IDRI, Lung Image Database Consortium and Image Database Resource Initiative; AIIB23, Airway-Informed Imaging Biomarker Challenge 2023; ISICDM, International Symposium on Image Computing and Digital Medicine; AeroPath, pathologically challenging airway dataset; RICORD, RSNA International COVID-19 Open Radiology Database; DLCST, Danish Lung Cancer Screening Trial; COPDGene, Genetic Epidemiology of Chronic Obstructive Pulmonary Disease study; COPD, chronic obstructive pulmonary disease; VESSEL12, VESsel SEgmentation in the Lung (2012 Challenge); SPIROMICS, SubPopulations and InteRmediate Outcome Measures in COPD Study; GOLD, Global Initiative for Chronic Obstructive Lung Disease; USA, United States of America.

References

[1] L. Dole, C.T. Mattos, J. Bianchi, H. Oh, K. Evangelista, J. Valladares Neto, S.L. Mota-Júnior, L. Cevidanes, J.C. Prieto, Enhancing airway obstruction diagnosis with multimodal 3D shape analysis, International Journal of Computer Assisted Radiology and Surgery (2025) 1–10.

[2] M. Mirus, E. Leitert, R. Bockholt, L. Heubner, S. Löck, M. Brei, J. Biehler, J.-P. Kühn, T. Koch, W. Wall, Radiomics-enhanced modelling approach for predicting the need for ECMO in ARDS patients: a retrospective cohort study, Scientific Reports 15 (2025) 34120.

[3] A.S.A. Rabby, M.F. Chaudhary, P. Saha, V. Sthanam, A. Nakhmani, C. Zhang, R.G. Barr, J. Bon, C.B. Cooper, J.L. Curtis, Light convolutional neural network to detect chronic obstructive pulmonary disease (COPDxNET): a multicenter model development and external validation study, medRxiv (2025).

[4] Z. Ma, Y. Sun, Z. Ma, L. Zhang, F. Cheng, H. Ma, L. Jin, M. Li, Chest CT imaging for differentiating normal, PRISm, and COPD in comparison with pulmonary function tests, La Radiologia Medica 130 (2025) 1786–1796.

[5] Z. Hu, T. Ren, M. Ren, W. Cui, E. Dong, P. Xue, A Precise Pulmonary Airway Tree Segmentation Method Using Quasi-Spherical Region Constraint and Tracheal Wall Gap Sealing, Sensors 24 (2024) 5104. https://doi.org/10.3390/s24165104.

[6] S. Wang, Y. Nan, S. Zhang, F. Felder, X. Xing, Y. Fang, J. Del Ser, S.L.F. Walsh, G. Yang, Probing perfection: The relentless art of meddling for pulmonary airway segmentation from HRCT via a human-AI collaboration based active learning method, Artificial Intelligence in Medicine 154 (2024) 102930. https://doi.org/10.1016/j.artmed.2024.102930.

[7] S. Ebrahimian, S.R. Digumarthy, B.C. Bizzo, K.J. Dreyer, M.K. Kalra, Automatic segmentation and measurement of tracheal collapsibility in tracheomalacia, Clinical Imaging 95 (2023) 47–51. https://doi.org/10.1016/j.clinimag.2022.11.020.

[8] W. Yu, H. Zheng, Y. Gu, F. Xie, J. Yang, J. Sun, G.-Z. Yang, TNN: Tree Neural Network for Airway Anatomical Labeling, IEEE Trans. Med. Imaging 42 (2023) 103–118. https://doi.org/10.1109/TMI.2022.3204538.

[9] A.P. Ratwani, H. Chen, L. Brown, E.A. Schwartz, K. Patel, A. Guttentag, T.A. McLaren, K.L. Sandler, O.B. Rickman, S. Shojaee, R.J. Lentz, F. Maldonado, Inter-rater reliability of a novel objective endpoint for benign central airway stenosis interventions: Segmentation-based volume rendering of computed tomography scans, PLoS ONE 18 (2023) e0290393. https://doi.org/10.1371/journal.pone.0290393.

[10] S.A. Nadeem, A.P. Comellas, E.A. Hoffman, P.K. Saha, Airway Detection in COPD at Low-Dose CT Using Deep Learning and Multiparametric Freeze and Grow, Radiology: Cardiothoracic Imaging 4 (2022) e210311. https://doi.org/10.1148/ryct.210311.

[11] H.-N. Cho, E. Gwon, K.-A. Kim, S.-H. Baek, N. Kim, S.-J. Kim, Accuracy of convolutional neural networks-based automatic segmentation of pharyngeal airway sections according to craniofacial skeletal pattern, American Journal of Orthodontics and Dentofacial Orthopedics 162 (2022) e53–e62. https://doi.org/10.1016/j.ajodo.2022.01.011.

[12] M.M. Boubnovski, M. Chen, K. Linton-Reid, J.M. Posma, S.J. Copley, E.O. Aboagye, Development of a multi-task learning V-Net for pulmonary lobar segmentation on CT and application to diseased lungs, Clinical Radiology 77 (2022) e620–e627. https://doi.org/10.1016/j.crad.2022.04.012.

[13] K. Orhan, M. Shamshiev, M. Ezhov, A. Plaksin, A. Kurbanova, G. Ünsal, M. Gusarev, M. Golitsyna, S. Aksoy, M. Mısırlı, F. Rasmussen, E. Shumilov, A. Sanders, AI-based automatic segmentation of craniomaxillofacial anatomy from CBCT scans for automatic detection of pharyngeal airway evaluations in OSA patients, Sci Rep 12 (2022) 11863. https://doi.org/10.1038/s41598-022-15920-1.

[14] I. Dudurych, A. Garcia-Uceda, Z. Saghir, H.A.W.M. Tiddens, R. Vliegenthart, M. De Bruijne, Creating a training set for artificial intelligence from initial segmentations of airways, Eur Radiol Exp 5 (2021) 54. https://doi.org/10.1186/s41747-021-00247-9.

[15] M. Zhang, C. Ding, S. Guo, Analysis of Tracheobronchial Diverticula Based on Semantic Segmentation of CT Images via the Dual-Channel Attention Network, Front. Public Health 9 (2022) 813717. https://doi.org/10.3389/fpubh.2021.813717.

[16] S. Shujaat, O. Jazil, H. Willems, A. Van Gerven, E. Shaheen, C. Politis, R. Jacobs, Automatic segmentation of the pharyngeal airway space with convolutional neural network, Journal of Dentistry 111 (2021) 103705. https://doi.org/10.1016/j.jdent.2021.103705.

[17] S. Ram, B.A. Hoff, A.J. Bell, S. Galban, A.B. Fortuna, O. Weinheimer, M.O. Wielpütz, T.E. Robinson, B. Newman, D. Vummidi, A. Chughtai, E.A. Kazerooni, T.D. Johnson, M.K. Han, C.R. Hatt, C.J. Galban, Improved detection of air trapping on expiratory computed tomography using deep learning, PLoS ONE 16 (2021) e0248902. https://doi.org/10.1371/journal.pone.0248902.

[18] R. Leonardi, A. Lo Giudice, M. Farronato, V. Ronsivalle, S. Allegrini, G. Musumeci, C. Spampinato, Fully automatic segmentation of sinonasal cavity and pharyngeal airway based on convolutional neural networks, American Journal of Orthodontics and Dentofacial Orthopedics 159 (2021) 824-835.e1. https://doi.org/10.1016/j.ajodo.2020.05.017.

[19] H.-H. Duan, J. Gong, X.-W. Sun, S.-D. Nie, Region growing algorithm combined with morphology and skeleton analysis for segmenting airway tree in CT images, Journal of X-Ray Science and Technology: Clinical Applications of Diagnosis and Therapeutics 28 (2020) 311–331. https://doi.org/10.3233/XST-190627.

[20] A.M. Fischer, A. Varga-Szemes, S.S. Martin, J.I. Sperl, P. Sahbaee, D. Neumann, J. Gawlitza, T. Henzler, C.M. Johnson, J.W. Nance, S.O. Schoenberg, U.J. Schoepf, Artificial Intelligence-based Fully Automated Per Lobe Segmentation and Emphysema-quantification Based on Chest Computed Tomography Compared With Global Initiative for Chronic Obstructive Lung Disease Severity of Smokers, Journal of Thoracic Imaging 35 (2020) S28–S34. https://doi.org/10.1097/RTI.0000000000000500.

[21] J. Park, J. Yun, N. Kim, B. Park, Y. Cho, H.J. Park, M. Song, M. Lee, J.B. Seo, Fully Automated Lung Lobe Segmentation in Volumetric Chest CT with 3D U-Net: Validation with Intra- and Extra-Datasets, J Digit Imaging 33 (2020) 221–230. https://doi.org/10.1007/s10278-019-00223-1.

[22] A. Khanna, N.D. Londhe, S. Gupta, Automated Localized Approach for Airway Segmentation in 3D Chest CT Volume, Biomed. Pharmacol. J. 13 (2020) 1671–1682. https://doi.org/10.13005/bpj/2042.

[23] A.M. Sousa, S.B. Martins, A.X. Falcão, F. Reis, E. Bagatin, K. Irion, ALTIS: A fast and automatic lung and trachea CT-image segmentation method, Medical Physics 46 (2019) 4970–4982. https://doi.org/10.1002/mp.13773.

[24] D. Gil, C. Sanchez, A. Borras, M. Diez-Ferrer, A. Rosell, Segmentation of distal airways using structural analysis, PLoS ONE 14 (2019) e0226006. https://doi.org/10.1371/journal.pone.0226006.

[25] R. Selvan, J. Petersen, J.H. Pedersen, M. De Bruijne, Extracting tree structures in CT data by tracking multiple statistically ranked hypotheses, Medical Physics 46 (2019) 4431–4440. https://doi.org/10.1002/mp.13711.

[26] H.B. Price, J.S. Kimbell, R. Bu, A.L. Oldenburg, Geometric Validation of Continuous, Finely Sampled 3-D Reconstructions From aOCT and CT in Upper Airway Models, IEEE Trans. Med. Imaging 38 (2019) 1005–1015. https://doi.org/10.1109/TMI.2018.2876625.

[27] J.-P. Charbonnier, E.M.V. Rikxoort, A.A.A. Setio, C.M. Schaefer-Prokop, B.V. Ginneken, F. Ciompi, Improving airway segmentation in computed tomography using leak detection with convolutional networks, Medical Image Analysis 36 (2017) 52–60. https://doi.org/10.1016/j.media.2016.11.001.

[28] B.C. Neelapu, O.P. Kharbanda, V. Sardana, A. Gupta, S. Vasamsetti, R. Balachandran, S.S. Rana, H.K. Sardana, A pilot study for segmentation of pharyngeal and sino-nasal airway subregions by automatic contour initialization, Int J CARS 12 (2017) 1877–1893. https://doi.org/10.1007/s11548-017-1650-1.

[29] H. Lim, O. Weinheimer, M.O. Wielpütz, J. Dinkel, T. Hielscher, D. Gompelmann, H.-U. Kauczor, C.P. Heussel, Fully Automated Pulmonary Lobar Segmentation: Influence of Different Prototype Software Programs onto Quantitative Evaluation of Chronic Obstructive Lung Disease, PLoS ONE 11 (2016) e0151498. https://doi.org/10.1371/journal.pone.0151498.

[30] I. Bernardeschi, D.D. Latta, G.L. Di Girolamo, V. Positano, M. Guadagni, M. Patronelli, A. Monteleone, D. Chiappino, An Operator Independent Method for Bronchial Tree Analysis from Trachea to the Small Airways Using Volumetric Multi-Detector Computed Tomography, J Med Imaging Hlth Inform 6 (2016) 170–176. https://doi.org/10.1166/jmihi.2016.1607.

[31] Z. Xu, U. Bagci, B. Foster, A. Mansoor, J.K. Udupa, D.J. Mollura, A hybrid method for airway segmentation and automated measurement of bronchial wall thickness on CT, Medical Image Analysis 24 (2015) 1–17. https://doi.org/10.1016/j.media.2015.05.003.

[32] J. Pu, C. Jin, N. Yu, Y. Qian, X. Wang, X. Meng, Y. Guo, A "loop" shape descriptor and its application to automated segmentation of airways from CT scans: A loop shape descriptor, Med. Phys. 42 (2015) 3076–3084. https://doi.org/10.1118/1.4921139.

[33] P. Nardelli, K.A. Khan, A. Corvò, N. Moore, M.J. Murphy, M. Twomey, O.J. O'Connor, M.P. Kennedy, R.S.J. Estépar, M.M. Maher, P. Cantillon-Murphy, Optimizing parameters of an open-source airway segmentation algorithm using different CT images, BioMed Eng OnLine 14 (2015) 62. https://doi.org/10.1186/s12938-015-0060-2.

[34] A. Fabijańska, Segmentation of pulmonary vascular tree from 3D CT thorax scans, Biocybernetics and Biomedical Engineering 35 (2015) 106–119. https://doi.org/10.1016/j.bbe.2014.07.001.

[35] C. Fetita, M. Ortner, P.-Y. Brillet, F. Preteux, P.A. Grenier, Volumetric Quantification of Airway Wall in CT via Collision-Free Active Surface Model: Application to Asthma Assessment, IEEE Trans. Med. Imaging 33 (2014) 1512–1526. https://doi.org/10.1109/TMI.2014.2316115.

[36] M. Heydarian, M.D. Noseworthy, M.V. Kamath, C. Boylan, W.F.S. Poehlman, A Morphological Algorithm for Measuring Angle of Airway Branches in Lung CT Images, Crit Rev Biomed Eng 42 (2014) 369–381. https://doi.org/10.1615/CritRevBiomedEng.2014012135.

[37] J. Petersen, M. Nielsen, P. Lo, L.H. Nordenmark, J.H. Pedersen, M.M.W. Wille, A. Dirksen, M. De Bruijne, Optimal surface segmentation using flow lines to quantify airway abnormalities in chronic obstructive pulmonary disease, Medical Image Analysis 18 (2014) 531–541. https://doi.org/10.1016/j.media.2014.02.004.

[38] V.R. De Water, J.K. Saridin, F. Bouw, M.M. Murawska, M.J. Koudstaal, Measuring Upper Airway Volume: Accuracy and Reliability of Dolphin 3D Software Compared to Manual Segmentation in Craniosynostosis Patients, Journal of Oral and Maxillofacial Surgery 72 (2014) 139–144. https://doi.org/10.1016/j.joms.2013.07.034.

[39] N. Mesanovic, H. Huseinagic, S. Mujagic, 3D TRACHEOBRONCHIAL AIRWAY TREE SEGMENTATION FROM THORAX CT IMAGES, Biomed. Eng. Appl. Basis Commun. 25 (2013) 1350015. https://doi.org/10.4015/S1016237213500154.

[40] X. Liu, D.Z. Chen, M.H. Tawhai, X. Wu, E.A. Hoffman, M. Sonka, Optimal Graph Search Based Segmentation of Airway Tree Double Surfaces Across Bifurcations, IEEE Trans. Med. Imaging 32 (2013) 493–510. https://doi.org/10.1109/TMI.2012.2223760.

[41] B. Lassen, E.M. Van Rikxoort, M. Schmidt, S. Kerkstra, B. Van Ginneken, J.-M. Kuhnigk, Automatic Segmentation of the Pulmonary Lobes From Chest CT Scans Based on Fissures, Vessels, and Bronchi, IEEE Trans. Med. Imaging 32 (2013) 210–222. https://doi.org/10.1109/TMI.2012.2219881.

[42] C.C. Chiang, M.N. Jeffres, A. Miller, D.C. Hatcher, Three-dimensional airway evaluation in 387 subjects from one university orthodontic clinic using cone beam computed tomography, The Angle Orthodontist 82 (2012) 985–992. https://doi.org/10.2319/122811-801.1.

[43] J. Pu, J.K. Leader, X. Meng, B. Whiting, D. Wilson, F.C. Sciurba, J.J. Reilly, W.L. Bigbee, J. Siegfried, D. Gur, Three-dimensional Airway Tree Architecture and Pulmonary Function, Academic Radiology 19 (2012) 1395–1401. https://doi.org/10.1016/j.acra.2012.06.007.

[44] T. Cheung, S. Oberoi, Three Dimensional Assessment of the Pharyngeal Airway in Individuals with Non-Syndromic Cleft Lip and Palate, PLoS ONE 7 (2012) e43405. https://doi.org/10.1371/journal.pone.0043405.

[45] R. Castillo, E. Castillo, M. McCurdy, D.R. Gomez, A.M. Block, D. Bergsma, S. Joy, T. Guerrero, Spatial correspondence of 4D CT ventilation and SPECT pulmonary perfusion defects in patients with malignant airway stenosis, Phys. Med. Biol. 57 (2012) 1855–1871. https://doi.org/10.1088/0031-9155/57/7/1855.

[46] E. Mortani Barbosa, G. Song, N. Tustison, M. Kreider, J.C. Gee, W.B. Gefter, D.A. Torigian, Computational Analysis of Thoracic Multidetector Row HRCT for Segmentation and Quantification of Small Airway Air Trapping and Emphysema in Obstructive Pulmonary Disease, Academic Radiology 18 (2011) 1258–1269. https://doi.org/10.1016/j.acra.2011.06.004.

[47] J. Petersen, M. Nielsen, P. Lo, Z. Saghir, A. Dirksen, M. De Bruijne, Optimal Graph Based Segmentation Using Flow Lines with Application to Airway Wall Segmentation, Information Processing in Medical Imaging 6801 (2011) 49–60. https://doi.org/10.1007/978-3-642-22092-0_5.

[48] Jiantao Pu, C. Fuhrman, W.F. Good, F.C. Sciurba, D. Gur, A Differential Geometric Approach to Automated Segmentation of Human Airway Tree, IEEE Trans. Med. Imaging 30 (2011) 266–278. https://doi.org/10.1109/TMI.2010.2076300.

[49] M.W. Graham, J.D. Gibbs, D.C. Cornish, W.E. Higgins, Robust 3-D Airway Tree Segmentation for Image-Guided Peripheral Bronchoscopy, IEEE Trans. Med. Imaging 29 (2010) 982–997. https://doi.org/10.1109/TMI.2009.2035813.

[50] P. Lo, B. Van Ginneken, J.M. Reinhardt, T. Yavarna, P.A. De Jong, B. Irving, C. Fetita, M. Ortner, R. Pinho, J. Sijbers, Extraction of airways from CT (EXACT'09), IEEE Transactions on Medical Imaging 31 (2012) 2093–2107.

[51] M. Zhang, Y. Wu, H. Zhang, Y. Qin, H. Zheng, W. Tang, C. Arnold, C. Pei, P. Yu, Y. Nan, G. Yang, S. Walsh, D.C. Marshall, M. Komorowski, P. Wang, D. Guo, D. Jin, Y. Wu, S. Zhao, R. Chang, B. Zhang, X. Lu, A. Qayyum, M. Mazher, Q. Su, Y. Wu, Y. Liu, Y. Zhu, J. Yang, A. Pakzad, B. Rangelov, R.S.J. Estepar, C.C. Espinosa, J. Sun, G.-Z. Yang, Y. Gu, Multi-site, Multi-domain Airway Tree Modeling, Medical Image Analysis 90 (2023) 102957. https://doi.org/10.1016/j.media.2023.102957.

[52] Y. Qin, H. Zheng, Y. Gu, X. Huang, J. Yang, L. Wang, Y.-M. Zhu, Learning bronchiole-sensitive airway segmentation CNNs by feature recalibration and attention distillation, in: Springer, 2020: pp. 221–231.

[53] Y. Nan, X. Xing, S. Wang, Z. Tang, F.N. Felder, S. Zhang, R.E. Ledda, X. Ding, R. Yu, W. Liu, F. Shi, T. Sun, Z. Cao, M. Zhang, Y. Gu, H. Zhang, J. Gao, P. Wang, W. Tang, P. Yu, H. Kang, J. Chen, X. Lu, B. Zhang, M. Mamalakis, F. Prinzi, G. Carlini, L. Cuneo, A. Banerjee, Z. Xing, L. Zhu, Z. Mesbah, D. Jain, T. Mayet, H. Yuan, Q. Lyu, A. Qayyum, M. Mazher, A. Wells, S.L. Walsh, G. Yang, Hunting imaging biomarkers in pulmonary fibrosis: Benchmarks of the AIIB23 challenge, Medical Image Analysis 97 (2024) 103253. https://doi.org/10.1016/j.media.2024.103253.

[54] W. Tan, P. Huang, X. Li, G. Ren, Y. Chen, J. Yang, Analysis of segmentation of lung parenchyma based on deep learning methods, Journal of X-Ray Science and Technology: Clinical Applications of Diagnosis and Therapeutics 29 (2021) 945–959. https://doi.org/10.3233/XST-210956.

[55] K.-H. Støverud, D. Bouget, A. Pedersen, H.O. Leira, T. Amundsen, T. Langø, E.F. Hofstad, AeroPath: An airway segmentation benchmark dataset with challenging pathology and baseline method, Plos One 19 (2024) e0311416.

[56] E.B. Tsai, S. Simpson, M.P. Lungren, M. Hershman, L. Roshkovan, E. Colak, B.J. Erickson, G. Shih, A. Stein, J. Kalpathy-Cramer, J. Shen, M. Hafez, S. John, P. Rajiah, B.P. Pogatchnik, J. Mongan, E. Altinmakas, E.R. Ranschaert, F.C. Kitamura, L. Topff, L. Moy, J.P. Kanne, C.C. Wu, The RSNA International COVID-19 Open Radiology Database (RICORD), Radiology 299 (2021) E204–E213. https://doi.org/10.1148/radiol.2021203957.

[57] S.G. Armato III, G. McLennan, L. Bidaut, M.F. McNitt-Gray, C.R. Meyer, A.P. Reeves, B. Zhao, D.R. Aberle, C.I. Henschke, E.A. Hoffman, The lung image database consortium (LIDC) and image database resource initiative (IDRI): a completed reference database of lung nodules on CT scans, Medical Physics 38 (2011) 915–931.

[58] J.H. Pedersen, H. Ashraf, A. Dirksen, K. Bach, H. Hansen, P. Toennesen, H. Thorsen, J. Brodersen, B.G. Skov, M. Døssing, The Danish randomized lung cancer CT screening trial—overall design and results of the prevalence round, Journal of Thoracic Oncology 4 (2009) 608–614.

[59] E.A. Regan, J.E. Hokanson, J.R. Murphy, B. Make, D.A. Lynch, T.H. Beaty, D. Curran-Everett, E.K. Silverman, J.D. Crapo, Genetic Epidemiology of COPD (COPDGene) Study Design, COPD: Journal of Chronic Obstructive Pulmonary Disease 7 (2011) 32–43. https://doi.org/10.3109/15412550903499522.

[60] R.D. Rudyanto, S. Kerkstra, E.M. Van Rikxoort, C. Fetita, P.-Y. Brillet, C. Lefevre, W. Xue, X. Zhu, J. Liang, İ. Öksüz, D. Ünay, K. Kadipaşaogˇlu, R.S.J. Estépar, J.C. Ross, G.R. Washko, J.-C. Prieto, M.H. Hoyos, M. Orkisz, H. Meine, M. Hüllebrand, C. Stöcker, F.L. Mir, V. Naranjo, E. Villanueva, M. Staring, C. Xiao, B.C. Stoel, A. Fabijanska, E. Smistad, A.C. Elster, F. Lindseth, A.H. Foruzan, R. Kiros, K. Popuri, D. Cobzas, D. Jimenez-Carretero, A. Santos, M.J. Ledesma-Carbayo, M. Helmberger, M. Urschler, M. Pienn, D.G.H. Bosboom, A. Campo, M. Prokop, P.A. De Jong, C. Ortiz-de-Solorzano, A. Muñoz-Barrutia, B. Van Ginneken, Comparing algorithms for automated vessel segmentation in computed tomography scans of the lung: the VESSEL12 study, Medical Image Analysis 18 (2014) 1217–1232. https://doi.org/10.1016/j.media.2014.07.003.

[61] D. Couper, L.M. LaVange, M. Han, R.G. Barr, E. Bleecker, E.A. Hoffman, R. Kanner, E. Kleerup, F.J. Martinez, P.G. Woodruff, S. Rennard, for the SPIROMICS Research Group, Design of the Subpopulations and Intermediate Outcomes in COPD Study (SPIROMICS): Table 1, Thorax 69 (2014) 492–495. https://doi.org/10.1136/thoraxjnl-2013-203897.
